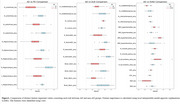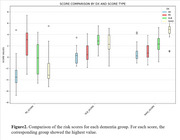# High‐order attention mechanism of MR imaging features predicts differential diagnosis for dementia

**DOI:** 10.1002/alz70856_106312

**Published:** 2026-01-09

**Authors:** WonJun Chun, Sung‐Woo Kim, Yeong‐Hun Song, Joon‐Kyung Seong, Min Seok Baek, Wha Jin Lee

**Affiliations:** ^1^ Korea University, Seoul, Korea, Republic of (South); ^2^ Wonju Severance Christian Hospital, Yonsei University Wonju College of Medicine, Wonju, Korea, Republic of (South); ^3^ NeuroXT, Seoul, Korea, Republic of (South)

## Abstract

**Background:**

Accurate differential diagnosis of dementia types is crucial for guiding therapeutic strategies. Previous studies using magnetic resonance imaging (MRI) for differential diagnosis focused on different patterns of cortical atrophy among dementia groups. In this study, we apply novel high‐order attention mechanism to classify four different diseases of dementia, of which low‐order attention was learned based on the task of classifying amyloid pathology in the spectrum of Alzheimer's disease. By leveraging an attention mechanism‐based deep learning model, we assess how non‐AD groups exhibit deviations in these predictive MRI features, enabling more precise differentiation of dementia subtypes.

**Method:**

We collected 99 T1‐weighted MRI images and demographic and clinical information from Wonju Severance Christian Hospital, including 24 subjects with AD (amyloid confirmed), 25 subjects each with Parkinson's Disease (PD), Dementia with Lewy Bodies (DLB), and subcortical vascular dementia (SVaD). We trained the low‐order attention‐based classifier for predicting global amyloid/tau positivity in the AD spectrum, using more than 12091 number of patient data from 5 datasets, including ADNI, HABS, and Korean datasets. The high‐order attention was then calculated based on the importance of each feature in estimating the probability of global amyloid/tau positivity. Using these differences as weights, we computed three non‐AD‐specific risk scores, defined as the weighted sum of feature importances (FI). A Support Vector Machine (SVM) classifier was then trained using five‐fold cross‐validation with these risk scores as inputs.

**Result:**

FIs of five features (e.g., entorhinal cortex, hippocampus) were significantly distinct in PD, six (e.g., ventral diencephalon, brainstem) in DLB, and eight (e.g., white matter hypointensities, choroid plexus) in SVaD (Figure 1). The obtained risk scores exhibit contrasts across diagnostic groups (Figure 1). For classification between AD and non‐AD groups, SVM achieved an average AUC of 0.96 ± 0.05, outperforming raw ROI volumes (AUC = 0.88 ± 0.11) or volume‐based risk scores (AUC = 0.91 ± 0.10).

**Conclusion:**

We propose novel differential scores based on AD signature features, incorporating high‐order attention mechanism learned from amyloid/tau pathology predicting model and inter‐feature relationships. The proposed high‐order attention mechanism enabled extraction of precise features with higher predictive power for AD pathologies, while capturing feature importances reflecting their mutual relationships.